# A New Family of Nonempirical Range-Separated Functionals: RSX‑r^2^SCAN, RSX‑r^2^SCAN0, and RSX‑r^2^SCAN-QIDH

**DOI:** 10.1021/acs.jctc.6c01471

**Published:** 2026-10-15

**Authors:** P. Maiz-Pastor, E. Brémond, A. J. Pérez-Jiménez, C. Adamo, J. C. Sancho-García

**Affiliations:** † Department of Physical Chemistry, 16718 University of Alicante, Alicante E-03080, Spain; ‡ 555089 Université Paris Cité, ITODYS, CNRS, Paris F-75006, France; § Institut Universitaire de France, 103 Boulevard Saint Michel, Paris F-75005, France; ∥ 52845 Chimie ParisTech, PSL Research University, CNRS, Institute of Chemistry for Life and Health Sciences (i-CLeHs), UMR 8060, Paris F-75005, France

## Abstract

We extend the restored-regularized Strongly Constrained and Appropriately Normed (r^2^SCAN) nonempirical exchange-correlation functional to the Range-Separation eXchange (RSX) scheme, RSX-r^2^SCAN, where the range-separation parameter (ω) is obtained by applying a first-principles constraint, thus not relying on any empirical fitting. The corresponding exchange-correlation hybrid (i.e., RSX-r^2^SCAN0) and double-hybrid (i.e., RSX-r^2^SCAN-QIDH) versions are also built following this procedure, concomitantly assessing all the models on relevant systems (e.g., increasingly larger clusters of noninteracting He atoms) and a large data set of ionization potentials (e.g., GW100). The assessment is also done using the GMTKN55 database for general main-group thermochemistry, kinetics, and noncovalent interactions, along with the WTMAD-2 error metric and a more recent one called WTMAD-4. The assessment is completed with a detailed analysis of some of the GMTKN55 subsets most affected by self-interaction errors. Finally, the models are coupled to the VV10 correlation energy functional (RSX-r^2^SCAN-NL, r^2^SCAN0-NL, and RSX-r^2^SCAN-QIDH-NL) for nonlocal and long-range electronic effects, to accurately address noncovalently bound systems as well.

## Graphical abstract

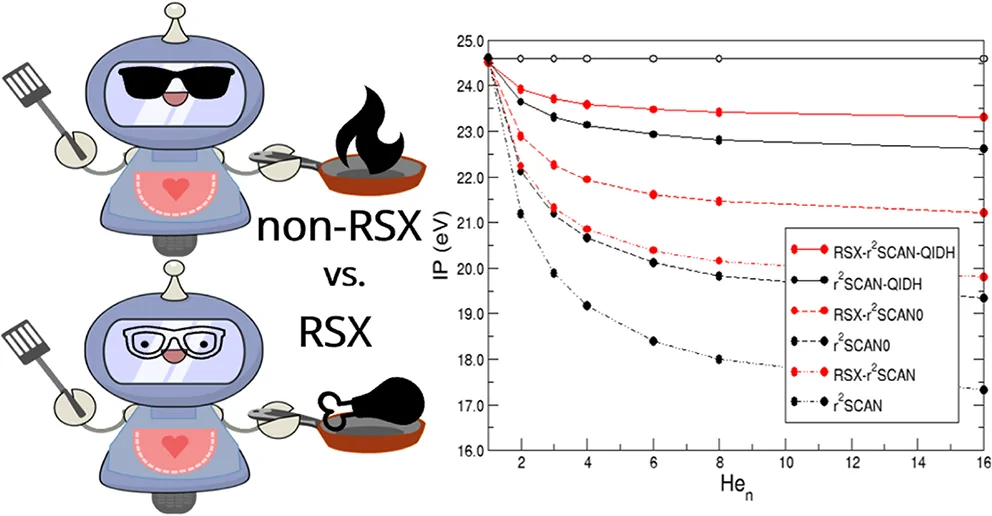

## 1 Introduction

The nonempirical SCAN (“strongly constrained and appropriately normed”) functional was developed^1^ in 2015 as a meta-GGA exchange-correlation model incorporating the correct uniform electron gas limit, proper behavior under density scaling, a large set of exact constraints on exchange and correlation energies, the exact exchange gradient expansion up to second order, among others, to name just a few of the exact physical and mathematical conditions imposed for its development. Later, the SCAN model evolved into the regularized SCAN (rSCAN) and finally into the restored-regularized (r^2^SCAN) versions^2,3^ retaining the main features of the underlying model while improving its numerical stability and transferability. Consequently, r^2^SCAN is almost unanimously considered one of the most successful developments so far for nonempirical density functional approximations (DFAs), thus preserving a large set of exact constraints and physical norms while introducing a carefully designed numerical regularization. This strategy is expected to lead to a separable functional that is both accurate and robust in practical calculations of any type:

1 $$E_{xc}^{r^{2}\mathrm{SCAN}}\left[\rho \left(r\right)\right]=E_{x}^{r^{2}\mathrm{SCAN}}\left[\rho \left(r\right)\right]+E_{c}^{r^{2}\mathrm{SCAN}}\left[\rho \left(r\right)\right]$$

Therefore, trying to respect as much as possible the rigorous nonempiricism of the whole model, in 2023 we benchmarked^4^ the corresponding hybrid (r^2^SCAN0) and double-hybrid (r^2^SCAN-QIDH) functionals, whose exchange and correlation components are defined as follows:

2 $$\begin{array}{ll} E_{xc}^{r^{2}\mathrm{SCAN}0}\left[\rho \left(r\right)\right]= & \frac{1}{4}E_{x}^{\mathrm{EXX}}\left[\phi_{i}\right]+\frac{3}{4}E_{x}^{r^{2}\mathrm{SCAN}}\left[\rho \left(r\right)\right]\\ & +E_{c}^{r^{2}\mathrm{SCAN}}\left[\rho \left(r\right)\right] \end{array}$$

3 $$\begin{array}{ll} E_{xc}^{r^{2}\mathrm{SCAN}-\mathrm{QIDH}}\left[\rho \left(r\right)\right]= & \frac{1}{3^{1/3}}E_{x}^{\mathrm{EXX}}\left[\phi_{i}\right]+\left(1-\frac{1}{3^{1/3}}\right)E_{x}^{r^{2}\mathrm{SCAN}}\left[\rho \left(r\right)\right]\\ & +\frac{1}{3}E_{c}^{\mathrm{PT}2}\left[\phi_{i},\phi_{a}\right]+\left(1-\frac{1}{3}\right)E_{c}^{r^{2}\mathrm{SCAN}}\left[\rho \left(r\right)\right] \end{array}$$

with the weights given to the different terms extracted from first-principles arguments,^5^ thus constituting a hierarchy of DFAs composed of meta-GGA (r^2^SCAN), hybrid meta-GGA (r^2^SCAN0) and double-hybrid meta-GGA (r^2^SCAN-QIDH) expressions. For the sake of comparison with other models, one can also define any hybrid (if *a*_*x*_ ≠ 0) and double-hybrid (if *a*_*x*_ ≠ 0 together with *a*_*c*_ ≠ 0) functional employing a general formulation:

4 $$E_{xc}\left[\rho \left(r\right)\right]=a_{x}E_{x}^{\mathrm{EXX}}\left[\phi_{i}\right]+\left(1-a_{x}\right)E_{x}\left[\rho \left(r\right)\right]+a_{c}E_{c}^{\mathrm{PT}2}\left[\phi_{i},\phi_{a}\right]+\left(1-a_{c}\right)E_{c}\left[\rho \left(r\right)\right]$$

with *a*_*x*_ and *a*_*c*_ the corresponding weights. The EXact-eXchange (EXX) term depends on the set of Kohn–Sham (KS) occupied orbitals ({ϕ_*i*_}) as does the second-order perturbation theory (PT2) term on the set of both occupied and virtual KS orbitals ({ϕ_*i*_,ϕ_*a*_}). We remark that the construction of any hybrid or double-hybrid functional does not, in general, preserve all the exact constraints satisfied by the parent semilocal functional, with some exceptions as the e.g., the PSTS hyper-GGA functional,^6^ which satisfies additional exact constraints but also requires some parameters. In this context, we emphasize that the numerical weights given above for the EXX and PT2 term (see eqs 2–3) were not obtained empirically but were based on theoretical constraints as well.^7–9^ For these reasons, we believe that this family of approximations deserves a nonempirical descriptor. Indeed, the whole hierarchy of models above can be considered nonempirical thanks to the combination of: (i) the r^2^SCAN functional incorporating, as emphasized before, the largest set of known exact constraints for an exchange-correlation expression; and (ii) a nonempirical framework to obtain the values of *a*_*x*_ and *a*_*c*_, weighting the $E_{x}^{\mathrm{EXX}}\left[\phi_{i}\right]$ and $E_{c}^{\mathrm{PT}2}\left[\phi_{i},\phi_{a}\right]$ energies, respectively, as remarked above.

This family of functionals (r^2^SCAN, r^2^SCAN0, and r^2^SCAN-QIDH) has been applied before^4^ to the large GMTKN55 data set,^10^ among other systems and data sets, showing robust accuracy compared with other expressions belonging to the same ladder of the hierarchy of methods. Other related (but parametrized) hybrid or spin-scaled double-hybrid versions of functionals based on r^2^SCAN have also been assessed in the literature.^4,11–14^ Taken into account this framework, our goal is thus to build here, in a nonempirical fashion, the range-separated version of the r^2^SCAN family of functionals. This would also allow us to compare the results with those obtained by using parametrized versions of range-separated expressions such as ωr^2^SCAN and ωPr^2^SCAN50, which are also available in the literature.^13^ Thus, while the building blocks are not new individually, that is, the r^2^SCAN exchange-correlation functional, the exchange range-separation, and, where applicable, the VV10 nonlocal correlation functional, their specific integration under a common minimally empirical strategy is, to the best of our knowledge, novel. More importantly, the resulting framework provides a systematic connection (i.e., a hierarchy of models) between semilocal, hybrid, and double-hybrid expressions, while retaining their minimally empirical character. Although empirical (parameter) optimization can improve statistical performance on a given benchmark set, a minimally empirical formulation remains equally valid from a theoretical standpoint, historically legitimate, and scientifically valuable. These formulations might provide a more direct assessment of the underlying physical model, avoiding potential overfitting.^15–18^

## 2 Theoretical and Computational Details

A well-established conceptual framework to improve the quality of any DFA, for certain systems and properties for which the asymptotic tail of the exchange-correlation potential is important, is to split the two-electron operator to guarantee the correct long-range behavior of the exchange potential. We choose here to respect the weight given to the hybrid functional at short range:^19^

5 $$\frac{1}{\left|r-r^{\prime }\right|}=\underset{\mathrm{short}-\mathrm{range}}{\underset{︸}{\frac{1-\left[\alpha +\beta \mathrm{erf}\left(\omega |r-r^{\prime }|\right)\right]}{\left|r-r^{\prime }\right|}}}+\underset{\mathrm{long}-\mathrm{range}}{\underset{︸}{\frac{\left[\alpha +\beta \mathrm{erf}\left(\omega |r-r^{\prime }|\right)\right]}{\left|r-r^{\prime }\right|}}}$$

with erf denoting the error function and α = *a*_*x*_, β = 1 – α, and ω defining the corresponding range-separation parameter. The determination of the range-separation parameter follows here a constraint-based approach, i.e., by enforcing that the considered DFA reproduces, as closely as possible, the exact total energy of the hydrogen atom, thus minimizing the deviation in the exact ground-state energy of the hydrogen atom with respect to the value of the range-separation (ω) introduced:

6 $$\omega \left(RSX\right)≔\underset{\omega \in R^{+}}{\min}\left|E_{exact}^{H-\mathrm{atom}}-E_{RSX-DFA}^{H-\mathrm{atom}}\left(\omega \right)\right|$$

until a residual error of less than 10^–6^ *E*_*h*_ is obtained. The above constraint for obtaining the value of ω can be viewed as a chemistry-driven condition, which has been applied previously by us (and others, such as the PBEmol^20^ or revTCA^21^ functionals) to some DFAs with considerable success for both ground-state^22–26^ and excited-state applications.^27,28^ Note that other physically motivated range-separation tuning strategies are also available in the literature,^29^ thus evidencing the interest in going beyond a simple parametrization. All the calculations were done using ORCA version 6.1.0,^30^ with a sufficiently large quadruple-ξ basis set (i.e., aug-cc-pVQZ^31^ or def2-QZVPP­(D)^32^) to avoid basis-set incompleteness issues, and employing dense numerical integration grids together with the resolution-of-the-identity approximation and the automatic generation of auxiliary basis sets^33^ technique.

Using this computational protocol and imposing the numerical condition given by eq 6, we determine the following range-separation values for ω: 0.209, 0.211, and 0.190 bohr^–1^ for RSX-r^2^SCAN, RSX-r^2^SCAN0, and RSX-r^2^SCAN-QIDH, respectively. We include for completeness in Table 1 the specific composition of all the models developed and assessed here. These values can be compared with the one previously obtained^13^ for ωr^2^SCAN (ω = 0.30 bohr^–1^) by optimizing its value to minimize the WTMAD (Weighted Total Mean Absolute Deviation) error of the GMTKN55 data set. We first note that the ω values (for r^2^SCAN-based functionals) are found to be much lower than those found previously for other range-separated DFAs, such as LC-ωPBE^34^ (ω = 0.47 bohr^–1^), but are comparable to those reported for the ωB97X family of functionals^35,36^ which were also empirically determined using a training set, with values for ω ranging from 0.2 to 0.3 bohr^–1^ depending on the particular DFA flavor chosen.

**Table 1:** Values of *a_x_, a_c_, c_ss_, c_os_, ω* (bohr^–1^), and b for All Density Functionals Considered in This Work

Functional	*a*_*x*_	*a*_*c*_	*c*_*os*_	*c*_*ss*_	ω	*b*
RSX-r^2^SCAN(-NL)	–	–	–	–	0.209	(11.05)
RSX-r^2^SCAN0(-NL)	1/4	–	–	–	0.211	(10.27)
RSX-r^2^SCAN-QIDH(-NL)	3^–1/3^	1/3	1	1	0.190	(20.90)
ωr^2^SCAN(-NL)^a^	–	–	–	–	0.300	(9.26)
ωr^2^SCANPr^2^SCAN50(-NL)^a^	1/2	7/20	4/3	0	0.214	(9.41)

a Taken from ref. 13.

We also notice that the ω value for this RSX-based set of DFAs varies only marginally around 0.200 bohr^–1^ with the global fraction of exact-like exchange in the chosen functional, whether it is semilocal, hybrid, or double hybrid. Actually, for an *ad hoc* value of $a_{x}=\frac{1}{2}$ (i.e., in a functional called r^2^SCANHH) a value of ω = 0.214 bohr^–1^ was found, being again very marginally different from the previous values. This is somehow different from what we found in previous investigations^23^ where the PBE exchange-correlation functional was used instead of r^2^SCAN, and might be interpreted as a sign of the more pronounced nonlocality of the r^2^SCAN-based exchange-correlation functional with respect to PBE. As we thoroughly compare along this work the RSX-based functionals with expressions already in the literature, such as the ωPr^2^SCAN50 double-hybrid functional, we finally comment on the meaning of the *c*_*ss*_ = 0 and *c*_*os*_ = 4/3 coefficients of that spin-scaled expression in Table 1, whose values arise from a previously disclosed relationship^37^ between the same-spin and opposite-spin contributions to the term $E_{c}^{\mathrm{PT}2}=c_{ss}E_{c}^{ss-\mathrm{PT}2}+c_{os}E_{c}^{os-\mathrm{PT}2}$ .

## 3 Results and Discussion

### 3.1 He*_n_* (Circular) Clusters of Increasing Size

We start probing the accuracy of the family of RSX-based functionals on a cluster of noninteracting He_*n*_ atoms (*n* = 1–16) regularly distributed on a planar circle; see Figure 1. The ionization potential (IP) for each value of *n* (i.e., as a function of the cluster size) must be equal to the IP of an individual He atom (taken here as 24.59 eV from the NIST database). The IP is calculated as IP­(Δ*S*CF) = *E*(*N* – 1) – *E*(*N*), with *E*(*N*) the energy of the neutral system and *E*(*N –* 1) the energy of the ionized system, all calculated with the very large aug-cc-pVQZ basis set. However, due to the delocalization error affecting, to a greater or lesser extent, all DFAs,^38–42^ the overstabilization of the energy of the positive hole created as *n* increases might lead to marked size-dependent deviations.^43^ The delocalization error (DE) will be thus calculated as DE = IP­(He*_n_*)_*n*=16_ – IP­(He_*n*_)_*n*=1_.

**Figure 1:**
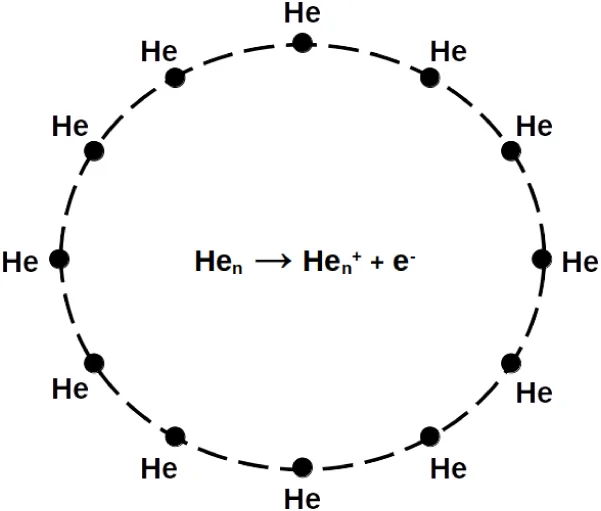
Sketch of the spatial arrangement of He*_n_* clusters (*n* = 1–16).

Figure 2 shows the evolution of the IP values as a function of *n*, for which large DE values of −7.29 and −5.24 eV are obtained, respectively, for semilocal (r^2^SCAN) and hybrid (r^2^SCAN0) functionals, in agreement with previous studies.^22^ This evolution is reminiscent of the different EXX weight (i.e., the value of *a*_*x*_) given to the DFA (see eqs 1–3) since EXX is by definition self-interaction-free. The application of the RSX partition to the r^2^SCAN-based versions of those functionals now led to a significant improvement in the results, with much lower DE values of −4.76 and −3.32 eV for RSX-r^2^SCAN and RSX-r^2^SCAN0. Other results available in the literature^22,43^ for various range-separated functionals, taken as examples, are −4.33, −3.51, and −3.20 eV for CAM-B3LYP, ωB97X, and LC-ωPBE, respectively, again signaling the need for range separation to obtain accurate results. For comparison, the parametrized ωr^2^SCAN gives a DE of −3.75 eV and is thus situated between that of RSX-r^2^SCAN and RSX-r^2^SCAN0.

**Figure 2:**
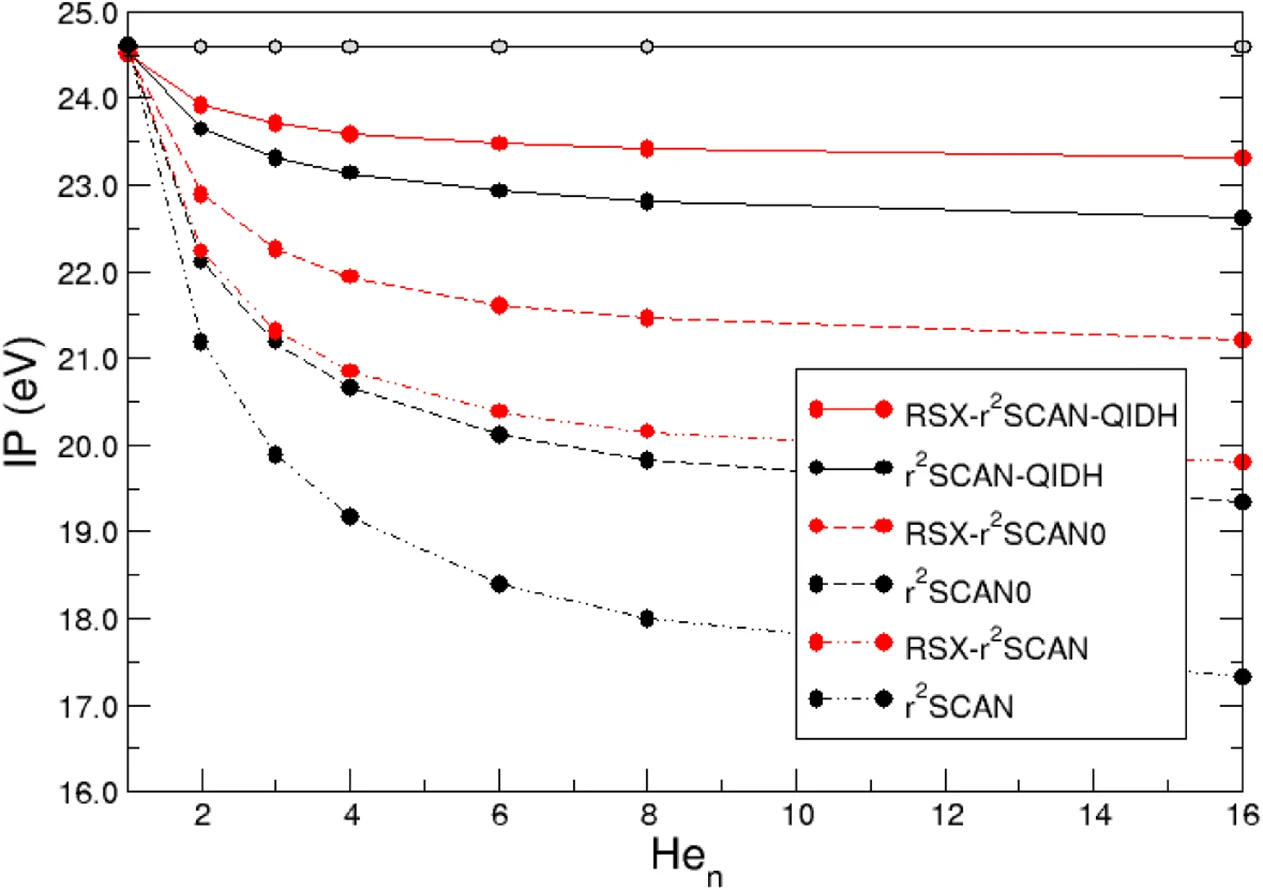
Evolution of IP values for He*_n_* clusters (*n* = 1–16) for r^2^SCAN-based exchange-correlation functionals, as a function of the system size *n*.

The use of a double-hybrid functional, such as r^2^SCAN-QIDH significantly improves the results, providing a much lower DE of −2.04 eV, again aligned with its higher EXX weight with respect to r^2^SCAN0 or r^2^SCAN. Remarkably, RSX-r^2^SCAN-QIDH leads to the lowest DE value of −1.19 eV among all the functionals considered here, while ωPr^2^SCAN50 gives a larger DE value of −2.39 eV. We also note in passing that the DE previously obtained^24^ at the RSX-PBE-QIDH level was as low as −0.92 eV. Overall, we clearly observe the large impact of the range-separation scheme on the results, independent of the underlying exchange-correlation functional (i.e., PBE or r^2^SCAN vs RSX-PBE or RSX-r^2^SCAN) or the composition of the double-hybrid functional (i.e., RSX-r^2^SCAN-QIDH vs ωPr^2^SCAN50).

Furthermore, we also inspect the values of the Highest Occupied Molecular Orbital (HOMO) for *n* = 16, or in other words, the accuracy of Koopmans’ theorem (IP ≈ −*ε*_*HOMO*_): values of −16.54, −18.73, and −22.57 eV are calculated for r^2^SCAN, r^2^SCAN0, and r^2^SCAN-QIDH, respectively, to be compared with values of −19.46, −20.98, and −23.40 eV when the RSX scheme is applied to the same functionals. Interestingly, a notable improvement is also found for all models compared with the reference value of 24.59 eV, even though the effect of the $E_{c}^{\mathrm{PT}2}$ term cannot be included during the self-consistent iterations, neither for r^2^SCAN-QIDH nor for RSX-r^2^SCAN-QIDH. This leads to a DE of 8.05, 5.82, and 2.02 eV for r^2^SCAN, r^2^SCAN0, and r^2^SCAN-QIDH, respectively, to be compared with 5.13, 3.61, and 1.19 eV for the corresponding RSX versions of those functionals. Note also that ωPr^2^SCAN50 provided a DE of 2.28 eV, which is higher than the best value obtained by RSX-r^2^SCAN-QIDH.

### 3.2 The GW100 Database

We now switch to the GW100 database of small molecules,^44^ for which we have computed (with the def2-QZVPP basis set) the vertical ionization potentials of all systems as IP­(Δ*S*CF) = *E*(*N –* 1) – *E*(*N*) at the reference DLPNO-CCSD­(T)/CBS­(3,4) level, i.e., the Domain-based Local Pair Natural Orbital technique applied to Coupled-Cluster Singles, Doubles, and perturbative Triples excitations, with the Complete Basis Set estimate using triple-ξ and quadruple-ξ basis sets.^45^ The GW100 data set covers a wide range of ionization potentials (i.e., from 4 eV for Rb_2_ to 25 eV for He) and chemical speciation (organic molecules, small metallic clusters, alkali metal halides, metal bonds, noble gases, etc.). Table 2 presents IP values, obtained as IP­(Δ*S*CF) = *E*(*N –* 1) – *E*(*N*), as well as the Mean Signed Deviation (MSD), Mean Absolute Deviation (MAD), and Root-Mean-Squared Deviation (RMSD) calculated for RSX-r^2^SCAN, RSX-r^2^SCAN0, and RSX-r^2^SCAN-QIDH.

**Table 2:** MSD, MAD, RMSD (All in eV), and Kurtosis Values for the IP of the GW100 Data Set of Compounds

	Functional	MSD	MAD	RMSD	Kurtosis
IP(Δ*S*CF)	RSX-r^2^SCAN	–0.178	0.214	0.323	0.66
	RSX-r^2^SCAN0	–0.138	0.179	0.241	0.74
	RSX-r^2^SCAN-QIDH	0.002	0.144	0.198	0.73
	ωr^2^SCAN	–0.070	0.136	0.216	0.63
	ωPr^2^SCAN50	–0.017	0.094	0.135	0.70
IP = −*ε*_HOMO_	RSX-r^2^SCAN	–1.176	1.181	1.458	0.81
	RSX-r^2^SCAN0	–0.581	0.605	0.817	0.74
	RSX-r^2^SCAN-QIDH	0.102	0.312	0.412	0.76
	ωr^2^SCAN	–0.459	0.539	0.813	0.66
	ωPr^2^SCAN50	–0.284	0.333	0.472	0.71

It is easy to observe that the MAD values consistently decrease as one ascends the hierarchy of methods, starting from a MAD of 0.21 eV for RSX-r^2^SCAN and reaching an error as low as 0.14 eV at the RSX-r^2^SCAN-QIDH level. We note in this case an improvement in RSX-r^2^SCAN-based functionals with respect to previous RSX-PBE-based results^24^ across all rungs of the hierarchy: (i) a MAD value of 0.27 eV for RSX-PBE vs 0.21 eV for RSX-r^2^SCAN; (ii) a MAD value of 0.25 eV for RSX-PBE0 vs 0.18 eV for RSX-r^2^SCAN0; and (iii) a MAD value of 0.24 eV for RSX-PBE-QIDH vs 0.14 eV for RSX-r^2^SCAN-QIDH. The use of the parametrized ωr^2^SCAN led to an even better value (calculated here) of 0.14 eV (see Table 2), which is further improved at the ωPr^2^SCAN50 level. Overall, we again observe the large impact of range separation on the accuracy of the results.

We next analyze the kurtosis value, which is defined as the MAD/RMSD ratio, used as a simple yet valuable statistical measure^46^ for the benchmarking of DFAs. A ratio around 0.8 (i.e., $\frac{MAD}{RMSD}=\sqrt{\frac{2}{\pi }}\approx 0.8$ for a normal distribution) indicates minor systematic errors, whereas a value below (above) 0.8 indicates a leptokurtic (platykurtic) distribution, which may indicate the presence of significant outliers (systematic bias). Table 2 shows slightly better values for RSX-r^2^SCAN (0.66) and RSX-r^2^SCAN-QIDH (0.73) compared with the corresponding values for ωr^2^SCAN (0.63) and ωPr^2^SCAN50 (0.70). However, this deviation from the ideal value does not seem to have any practical significance if one looks at the good correlation found between the reference [i.e., DLPNO-CCSD­(T)/CBS­(3,4)] and the calculated values (see Figure 3). Actually, for all the methods assessed here we can observe an almost perfect correlation (*r* ^2^ ≥ 0.996) between the two sets of values, with the slopes of the linear regression ranging from 0.980 (for RSX-r^2^SCAN) to 0.998–0.999 for the rest of the models.

**Figure 3:**
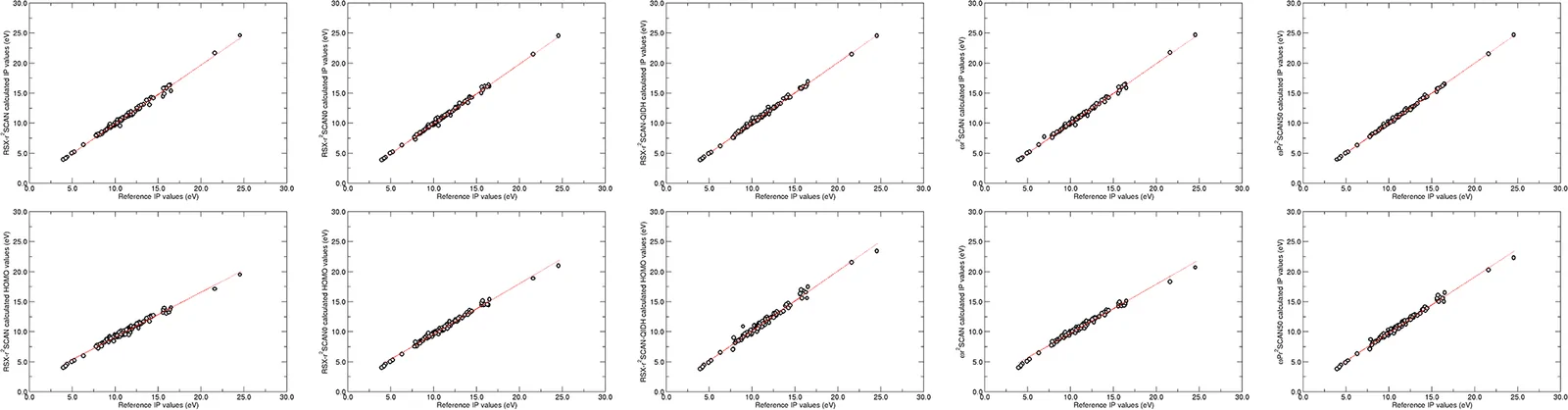
Top: Correlation diagrams comparing the reference and calculated ionization potentials for the GW100 data set (from left to right) calculated at the RSX-r^2^SCAN, RSX-r^2^SCAN0, RSX-r^2^SCAN-QIDH, ωr^2^SCAN, and ωPr^2^SCAN50 levels. Bottom: Correlation diagrams comparing the reference ionization potentials and calculated HOMO energies for the GW100 data set (from left to right) calculated at the RSX-r^2^SCAN, RSX-r^2^SCAN0, RSX-r^2^SCAN-QIDH, ωr^2^SCAN, and ωPr^2^SCAN50 levels.

Additionally, we compare in Figure 3 the (ideally linear) correlation between the reference IP values and those obtained from the energy of the HOMO orbital, and we observe a progressive improvement going from RSX-r^2^SCAN (slope: 0.75) to RSX-r^2^SCAN0 (slope: 0.84) to RSX-r^2^SCAN-QIDH (slope: 1.00). The same degree of improvement is also found for ωPr^2^SCAN50 (slope: 0.93) compared to ωr^2^SCAN (slope: 0.81), although with slightly less accurate values than RSX-r^2^SCAN-QIDH. This trend is corroborated by the MSD, MAD, and RMSD reported in Table 2, with RSX-r^2^SCAN-QIDH confirmed as the most accurate functional here.

### 3.3 The GMTKN55 Database and Its Subsets

#### 3.3.1 Analysis of Some Key Subsets

Before commenting on the results for the whole GMTKN55 database, we will first inspect some subsets that are closely related to the previously described properties and are thus expected to be more affected by using a functional with exchange range separation. Therefore, the assessment is detailed first for (vertical) ionization potentials of the G21IP subset, comprising 36 molecules, yielding MAD values of 4.65, 4.72, and 3.37 kcal mol^–1^ at the RSX-r^2^SCAN, RSX-r^2^SCAN0, and RSX-r^2^SCAN-QIDH levels, respectively, which are similar to those found for the corresponding r^2^SCAN, r^2^SCAN0, and r^2^SCAN-QIDH functionals (4.69, 4.81, and 3.12 kcal mol^–1^). These values can also be compared with those achieved previously by other range-separated models, such as ωB97X-V or ωB97X-D3 leading^10^ to (roughly speaking) 2.9 kcal mol^–1^, or with the MAD value provided by the parametrized ωr^2^SCAN model (5.13 kcal mol^–1^).

We next peruse the DIPCS10 subset of double ionization potentials, for which the set of RSX-based models led to the following results with respect to the corresponding pristine expressions: MAD values of 4.66 vs 5.12 kcal mol^–1^ (RSX-r^2^SCAN vs r^2^SCAN), 4.72 vs 4.58 kcal mol^–1^ (RSX-r^2^SCAN0 vs r^2^SCAN0), and 3.45 vs 3.39 kcal mol^–1^ (RSX-r^2^SCAN-QIDH vs r^2^SCAN-QIDH). For the sake of completeness, we again looked at the performance of other range-separated DFAs, such as ωB97X-V (MAD of 4.10 kcal mol^–1^), ωB97X-D3 (MAD of 5.45 kcal mol^–1^), and ωr^2^SCAN (MAD of 7.10 kcal mol^–1^).

The assessment of the RSX-based models for (vertical) electron affinities, calculated as EA­(Δ*S*CF) = *E*(*N*) – *E*(*N* + 1), will next be performed by considering the G21EA subset (composed of 25 molecules) as a test case, which is also part of the GMTKN55 database. The MAD values of r^2^SCAN, r^2^SCAN0, and r^2^SCAN-QIDH (3.87, 5.01, and 3.77 kcal mol^–1^, respectively) are larger than the results obtained when the RSX scheme is applied, leading to MAD values of 3.53, 3.84, and 3.54 kcal mol^–1^ for RSX-r^2^SCAN, RSX-r^2^SCAN0, and RSX-r^2^SCAN-QIDH, respectively. These values are still larger than the MAD results achieved by other range-separated functionals such as ωB97X-V or ωB97X-D3, with MADs of 1.85 and 1.53 kcal mol^–1^, respectively, but comparable to or even slightly smaller than those provided by the parametrized ωr^2^SCAN (MAD of 3.76 kcal mol^–1^).

We also analyze the PA26 subset (comprising 26 molecules) of proton affinities, which is also part of the GMTKN55 database, for which RSX-based models led to a large improvement with respect to non-RSX models: MAD values of 1.55 vs 2.51 kcal mol^–1^ (RSX-r^2^SCAN vs r^2^SCAN) and 1.47 vs 2.53 kcal mol^–1^ (RSX-r^2^SCAN0 vs r^2^SCAN0). The use of a double-hybrid version (RSX-r^2^SCAN-QIDH vs r^2^SCAN-QIDH) did not significantly alter the values: 1.24 vs 1.38 kcal mol^–1^, respectively. These values can also be compared to the MAD value of 3.66 kcal mol^–1^ at the ωB97X-D3 level. The application of ωr^2^SCAN also gave a low MAD value of 1.30 kcal mol^–1^, as reported in ref. 12.

We also separately analyzed the SIE4 × 4 subset, composed of a set of reaction energies surveying the dissociation of cationic systems, and thus prone to marked self-interaction errors. Standard semilocal and hybrid DFAs are known to give large errors, as is also the case for r^2^SCAN and r^2^SCAN0, for which MAD values as large as 18.06 and 10.87 kcal mol^–1^, respectively, are calculated. The corresponding MAD values for RSX-r^2^SCAN and RSX-r^2^SCAN0 are consistently reduced to 15.40 and 8.98 kcal mol^–1^, and thus become comparable to those achieved by other range-separated models such as ωB97X-V (11.49 kcal mol^–1^) or ωB97X-D3 (12.35 kcal mol^–1^) taken from ref. 10, or by the parametrized ωr^2^SCAN model with a MAD value of 11.53 kcal mol^–1^. However, the application of a double-hybrid model such as r^2^SCAN-QIDH dramatically improves the results, providing a MAD of 2.68 kcal mol^–1^ which is further reduced to 2.21 kcal mol^–1^ for the corresponding RSX-r^2^SCAN-QIDH expression.

The above analysis indicates that going from an RSX-based meta-GGA (RSX-r^2^SCAN) to an RSX-based hybrid meta-GGA (RSX-r^2^SCAN0) is not as important as going a step further within the hierarchy of functionals (RSX-r^2^SCAN-QIDH). Therefore, since, within this hierarchy of RSX-r^2^SCAN-based functionals, the lowest MAD values are consistently provided by the RSX-r^2^SCAN-QIDH model, we will next compare in more detail its performance with respect to the corresponding (but parametrized) ωPr^2^SCAN50 double-hybrid version developed in ref. 12. Looking again at the subsets G21IP, DIPCS10, G21EA, and PA26, the MAD values of ωPr^2^SCAN50 (2.47, 2.18, 1.78, and 0.83 kcal mol^–1^, respectively) are lower than those provided by RSX-r^2^SCAN-QIDH (3.37, 3.45, 3.54, and 1.24 kcal mol^–1^, respectively). However, RSX-r^2^SCAN-QIDH significantly improves the MAD for the SIE4 × 4 data set, with a MAD value of 2.21 kcal mol^–1^ compared to 6.16 kcal mol^–1^ given by ωPr^2^SCAN50.

We finally look at the MB16-43 data set since it has been recently identified^47^ as a key subset for the design and transferability principles affecting any DFA. This data set is the only one in GMTKN55 whose chemical structures are not designed by humans and are instead “mindlessly” generated. First of all, to adequately bracket the results calculated here, we recall that the MAD values for the r^2^SCAN-based hierarchy of models are known^4^ to be r^2^SCAN (12.7 kcal mol^–1^), r^2^SCAN0 (13.9 kcal mol^–1^), r^2^SCAN0-DH (14.4 kcal mol^–1^), r^2^SCAN-QIDH (16.2 kcal mol^–1^), and r^2^SCAN0-2 (17.1 kcal mol^–1^), which in fact evolve as a function of two factors: i.e., the ladder itself, going from meta-GGA (r^2^SCAN) to hybrid (r^2^SCAN0) or double-hybrid (r^2^SCAN0-DH, r^2^SCAN-QIDH, and r^2^SCAN0-2) models, and the %EXX, i.e., the *a*_*x*_ in eq 4, introduced into the latter (50% for r^2^SCAN0-DH, 69% for r^2^SCAN-QIDH, and 79% for r^2^SCAN0-2). If we analyze now the MAD values provided by RSX-r^2^SCAN, RSX-r^2^SCAN0, and RSX-r^2^SCAN-QIDH (15.1, 18.7, and 17.8 kcal mol^–1^, respectively), we can observe a deterioration of the results (between 3 and 5 kcal mol^–1^) when comparing the same level of Jacob’s ladder. Note that the same deterioration also occurs, but much more markedly, for ωr^2^SCAN (20.8 kcal mol^–1^), with ωPr^2^SCAN50 (12.6 kcal mol^–1^) thus being more accurate than the rest of the models. In any case, these values are considerably lower than those provided by ωB97X-D3 (36.5 kcal mol^–1^) or ωB97X-V (32.5 kcal mol^–1^) or other double-hybrid functionals without range separation (e.g., B2-PLYP or B2GP-PLYP with MAD values of 34.4 and 26.1 kcal mol^–1^, respectively). Looking at the kurtosis value for this delicate MB16-43 data set, we observe values close to 0.8 for all the functionals analyzed here (0.77 and 0.82 for RSX-r^2^SCAN and RSX-r^2^SCAN-QIDH, or 0.78 and 0.81 for ωr^2^SCAN and ωPr^2^SCAN50, respectively).

#### 3.3.2 The Whole GMTKN55 Database

Having looked in detail at some key subsets of the GMTKN55 database, Table 3 now presents the results for the whole database (1505 relative energies) comprising basic properties and reaction energies for small systems (subsets W4-11, G21EA, G21IP, DIPCS10, PA26, SIE4 × 4, ALKBDE10, YBDE18, AL2 × 6, HEAVYSB11, NBPRC, ALK8, RC21, G2RC, BH76RC, FH51, TAUT15, and DC13), isomerization and reaction energies for large systems (subsets MB16-43, DARC, RSE43, BSR36, CDIE20, ISO34, ISOL24, C60ISO, and PArel), reaction barrier heights (subsets BH76, BHPERI, BHDIV10, INV24, BHROT27, PX13, and WCPT18), and intramolecular (subsets IDISP, ICONF, ACONF, Amino20 × 4, PCONF21, MCONF, SCONF, UPU23, and BUT14DIOL) and intermolecular (subsets RG18, ADIM6, S22, S66, HEAVY28, WATER27, CARBHB12, PNICO23, HAL59, AHB21, CHB6, and IL16) noncovalent interactions. We will also refer separately hereafter to these families of data points, commonly abbreviated as “basic + small”, “iso. + large”, “barriers”, “intramol. NCI”, and “intermol. NCI”. We will first rely on the WTMAD-2 (Weighted Total Mean Absolute Deviation) metric, for the whole database or for the above families of properties, defined as

$$WTMAD-2=\frac{1}{N_{total}}\overset{N_{subsets}}{\underset{j}{\sum }}N_{j}\frac{{\overset{―}{\left|\Delta E\right|}}_{total}}{{\overset{―}{\left|\Delta E\right|}}_{j}}{MAD}_{j}$$ 7

where the sum extends (for the GMTKN55) over the total number of subsets (*N*_*subsets*_ = 55) with *N*_*j*_ energies, and ${\overset{―}{\left|\Delta E\right|}}_{j}$ the average absolute (reference) energy of the *j*th subset with a MAD of MAD_*j*_. Furthermore, $N_{total}=\overset{N}{\underset{j}{\sum }}N_{j}$ is the total number of data points (1505), and ${\overset{―}{\left|\Delta E\right|}}_{total}$ is the average of all ${\overset{―}{\left|\Delta E\right|}}_{j}$ values.

**Table 3:** WTMAD-2A^a^ (in kcal mol^–1^) for the Different Subsets and the Global GMTKN55 Database

		basic +	iso. +			
Functional	small	large	barriers	intramol. NCI	intermol. NCI	GMTKN55
RSX-r^2^SCAN	4.75	7.71	11.87	11.52	11.24	8.77
RSX-r^2^SCAN-NL	4.95	6.68	12.90	8.16	7.10	7.31
RSX-r^2^SCAN0	4.88	8.03	6.76	11.32	11.51	8.21
RSX-r^2^SCAN0-NL	5.15	6.85	7.64	7.04	6.39	6.36
RSX-r^2^SCAN-QIDH	4.04	6.12	4.39	5.81	6.39	5.24
RSX-r^2^SCAN-QIDH-NL	4.13	5.89	4.56	5.11	5.08	4.85
ωr^2^SCAN	4.26	7.50	7.69	12.03	12.85	8.46
ωr^2^SCAN-NL^b^	4.37	5.89	8.11	6.53	5.49	5.74
ωPr^2^SCAN50	2.40	4.35	3.68	6.40	8.20	4.83
ωPr^2^SCAN50-NL^b^	2.51	3.25	4.31	3.51	3.68	3.29

a Calculated with the ${\overset{―}{\left|\Delta E\right|}}_{total}=57.817\;\mathrm{kcal}\;{\mathrm{mol}}^{-1}$ value for each family and for the whole GMTKN55 database.

b Taken from ref. 13.

The value used for ${\overset{―}{\left|\Delta E\right|}}_{total}$ has also been a source of differences between the studies reported in the literature. First, a value of ${\overset{―}{\left|\Delta E\right|}}_{total}=56.84\mathrm{kcal}{\mathrm{mol}}^{-1}$ was so far used^10,48^ but it was later corrected^13^ to ${\overset{―}{\left|\Delta E\right|}}_{total}=57.817$ kcal mol^–1^, which will also be used here to facilitate comparison with related work. Additionally, one might not use the same value of ${\overset{―}{\left|\Delta E\right|}}_{total}$ for each of the families into which the whole GMTKN55 database is decomposed, given the large differences in the relative energies associated with each of the subsets of the families and between the families themselves, but rather a specific value (kcal mol^–1^) for each one: 111.49 for “basic + small”, 74.23 for “iso. + large”, 27.33 for “barriers”, 4.61 for “intramol. NCI”, and 22.69 for “intermol. NCI”. We will refer to these two approaches as WTMAD-2A and WTMAD-2B, respectively.

First of all, the results (WTMAD-2) in Table 3 for RSX-r^2^SCAN and ωr^2^SCAN are comparable for the whole GMTKN55 database (8.8 vs 8.5 kcal mol^–1^, respectively), but not for many of the individual subsets (see the Supporting Information) which might differ considerably. Table 3 also shows that RSX-r^2^SCAN0 is not a considerable improvement over RSX-r^2^SCAN, considering not only the global WTMAD-2 value of 8.2 vs 8.8 kcal mol^–1^ but also the values for each of the families comprising the GMTKN55 database, except for the “barriers” family of subsets, for which a large reduction (11.9 vs 6.8 kcal mol^–1^) is found for RSX-r^2^SCAN0. As might also be expected from the previous analysis of some key subsets, the range-separated double-hybrid functionals RSX-r^2^SCAN-QIDH and ωPr^2^SCAN50 lead to the lowest WTMAD-2 values (5.2 and 4.8 kcal mol^–1^, respectively) and do not differ much from each other (1 −2 kcal mol^–1^) for the different families of subsets. The RSX-r^2^SCAN-QIDH functional improves the WTMAD-2A for intra- and intermolecular noncovalent interactions, while ωPr^2^SCAN50 improves the other families. Table 4 presents the corresponding WTMAD-2B values, showing the same trends disclosed before, and thus will not be further analyzed.

**Table 4:** WTMAD-2B^a^ (in kcal mol^–1^) for the Different Subsets and the Global GMTKN55 Database

		basic +	iso. +			
Functional	small	large	barriers	intramol. NCI	intermol. NCI	GMTKN55
RSX-r^2^SCAN	9.16	9.90	5.61	0.92	4.41	8.77
RSX-r^2^SCAN-NL	9.55	8.57	6.09	0.65	2.79	7.31
RSX-r^2^SCAN0	9.41	10.31	3.19	0.90	4.52	8.21
RSX-r^2^SCAN0-NL	9.92	8.80	3.61	0.56	2.51	6.36
RSX-r^2^SCAN-QIDH	7.78	7.86	2.08	0.46	2.51	5.24
RSX-r^2^SCAN-QIDH-NL	7.96	7.56	2.15	0.41	2.00	4.85
ωr^2^SCAN	8.21	9.63	3.64	0.96	5.04	8.46
ωr^2^SCAN-NL^b^	8.42	7.56	3.84	0.52	2.14	5.74
ωPr^2^SCAN50	4.64	5.59	1.74	0.51	3.22	4.83
ωPr^2^SCAN50-NL^b^	4.84	4.17	2.03	0.28	1.44	3.29

a Calculated with ${\overset{―}{\left|\Delta E\right|}}_{family}$ specific to each family and with the ${\overset{―}{\left|\Delta E\right|}}_{total}=57.817\;\mathrm{kcal}{\;\mathrm{mol}}^{-1}$ value for the whole GMTKN55 database.

b Taken from ref. 13.

Finally, we have compared the performance of RSX-r^2^SCAN-QIDH and r^2^SCAN-QIDH across the GMTKN55 database, see Figure 4. As we can easily see from this graphical comparison, both functionals behave quite similarly for the GMTKN55 database, with the largest deviation being (in absolute value) 1.6 kcal mol^–1^ for the MB16-43 subset, favoring the r^2^SCAN-QIDH version in this case. On the other hand, the improvement of RSX-r^2^SCAN-QIDH with respect to the corresponding non-RSX version amounts to 0.5–0.7 kcal mol^–1^ for the ALK8, BHPERI, or SIE4 × 4 subsets. Overall, the WTMAD-2 and WTMAD-4 values for r^2^SCAN-QIDH are 4.56 and 5.61 kcal mol^–1^, respectively, and are thus slightly lower than those provided by the RSX-r^2^SCAN-QIDH functional.

**Figure 4:**
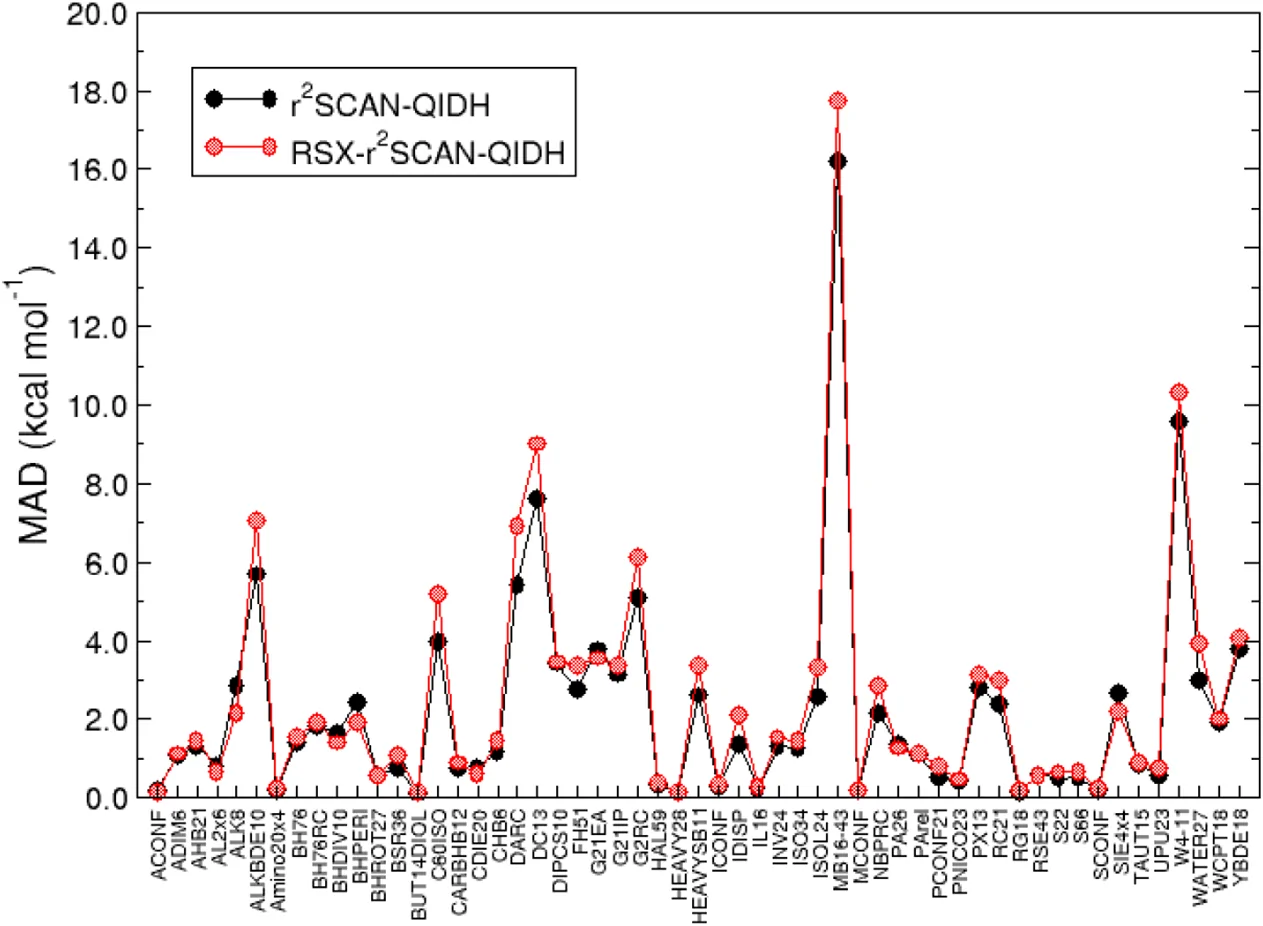
The MAD for each subset of the GMTKN55 database: RSX-r^2^SCAN-QIDH vs r^2^SCAN-QIDH.

#### 3.3.3 Combination with Dispersion Corrections: RSX-r^2^SCAN-NL, RSX-r^2^SCAN0-NL, and RSX-r^2^SCAN-QIDH-NL Functionals

We next couple the VV10 nonlocal correlation functional^49^ to the RSX-r^2^SCAN, RSX-r^2^SCAN0, and RSX-r^2^SCAN-QIDH models to also capture the nonlocal nature of the correlation forces through instantaneous and fluctuating dipole moments at spatially separated points **r** and **r**’:

8 $$E_{c}^{\mathrm{VV}10}\left[\rho \left(r\right),\rho \left(r^{\prime }\right)\right]=\int dr\rho \left(r\right)\left[\beta \left(b\right)+\frac{1}{2}\int dr^{\prime }\Phi \left(b,r,r^{\prime }\right)\rho \left(r^{\prime }\right)\right]$$

where β­(*b*) is a function of the parameter *b*, used previously to efficiently merge all the correlation energy terms entering into the final expression.^50–52^ The function Φ­(*b*,**r**,**r**’) is the correlation kernel of the electron densities, and also depends on *b*, behaving as $\propto \frac{1}{|r-r^{\prime }{|}^{6}}$ and thus decaying asymptotically correctly. We are also aware of other existing successful expressions for correcting a functional for dispersion interactions, such as e.g., D4,^53^ but we choose here the VV10 correlation functional instead to better preserve the minimally empirical nature of the whole models.^54^

We obtain the value of *b* (see Table 1) by minimizing the WTMAD-2B error (the value of *b* does not change if WTMAD-2A is chosen instead) for the group of subsets (RG18, ADIM6, S22, S66, HEAVY28, WATER27, CARBHB12, PNICO23, HAL59, AHB21, CHB6, and IL16), thus constituting the intermolecular noncovalent-interaction portion of the GMTKN55 database. The performance of the dispersion-corrected RSX-r^2^SCAN-NL, RSX-r^2^SCAN0, and RSX-r^2^SCAN-QIDH-NL functionals can be observed in Table 3 (WTMAD-2A) and Table 4 (WTMAD-2B), which, as expected, improve the subsets comprising the intra- and intermolecular noncovalent-interaction families of the GMTKN55 database, together with other subsets related to isomerization energies. The effect on the semilocal and hybrid models (comparing RSX-r^2^SCAN vs RSX-r^2^SCAN-NL, and RSX-r^2^SCAN0 vs RSX-r^2^SCAN0-NL) is more pronounced, as also happens when comparing ωr^2^SCAN and ωr^2^SCAN-NL, whereas the effect for the corresponding double-hybrid versions is attenuated, which is attributed to the partial introduction of noncovalent interactions by the PT2 term.

As a final test case for the RSX-r^2^SCAN-QIDH-NL functional we employ the NCIBLIND10 ^55^ and S22 × 5 ^56^ data sets. The former is composed of 10 dimers ranging from 6 to 32 atoms, at different separations between the centers of mass of the monomers, while the latter is an extension of the S22 data set for nonequilibrium geometries, thus representing systems outside the training set for coupling the VV10 and RSX-r^2^SCAN-QIDH functionals. Note that nonequilibrium geometries are always a difficult situation for the benchmarking of functionals and dispersion corrections.^57^ For the NCIBLIND10 data set of weakly interacting dimers, RSX-r^2^SCAN-QIDH leads to MAD and RMSD values (all in kcal mol^–1^) of 0.249 and 0.460, which slightly reduce to 0.223 and 0.440 for RSX-r^2^SCAN-QIDH-NL. The application of the RSX-r^2^SCAN-QIDH functional to the S22 × 5 data set leads to MAD and RMSD values (all in kcal mol^–1^) of 0.513 and 0.809, with the effect of the dispersion correction leading to lower MAD and RMSD values of 0.379 and 0.713 eV, respectively.

#### 3.3.4 WTMAD-2 vs WTMAD-4

Given the disparities previously found for WTMAD-2, not only due to the different values of ${\overset{―}{\left|\Delta E\right|}}_{total}$ previously used in the literature but also because each subset does not contribute evenly to the overall value, Bryenton and Johnson recently reformulated WTMAD-2 into the WTMAD-4 expression:^58^

$$WTMAD-4=\frac{1}{N_{total}}\overset{N_{subsets}}{\underset{j}{\sum }}w_{j}{MAD}_{j}$$ 9

where the weight given to each of the subsets is

10 $$w_{j}=\frac{100}{N_{subsets}}\left(\frac{3.5}{\overset{―}{{MAD}_{j}}}\right)$$

with *N*_*subsets*_ = 55 being the number of subsets and MAD_*j*_ is the MAD value for each of the *j* subsets. However, $\overset{―}{{MAD}_{j}}$ is here the mean MAD for subset *j* is taken over a set of ten functionals selected by the developers of this metric [i.e., PBE0-D3­(BJ), B3LYP-D3­(BJ), B3P86-D3­(BJ), B3PW91-D3­(BJ), PW1PW91-D3(0), MPW1PW91-D3­(BJ), BHLYP-D3­(BJ), HSE06-D3­(BJ), HISS-D3­(BJ), and LC-ωhPBE-D3­(BJ)]. Note that both WTMAD-2 and WTMAD-4 are global performance metrics for the GMTKN55 benchmark suite, but they differ in how the individual benchmark subsets are weighted, with the latter ensuring that all benchmark subsets contribute more equitably to the global value.

Table 5 shows the WTMAD-4 values for the set of functionals developed here. RSX-r^2^SCAN now performs better than ωr^2^SCAN for the whole GMTKN55 database (9.0 vs 9.7 kcal mol^–1^) and for most of its families. This performance is improved upon adding the nonlocal correlation functional to both models, RSX-r^2^SCAN-NL and ωr^2^SCAN-NL, decreasing the WTMAD-4 for both by approximately 2–3 kcal mol^–1^ thanks to the large improvement obtained for the subsets defining the intra- and intermolecular noncovalent interactions. The WTMAD-4 value of 6–7 kcal mol^–1^ provided by ωr^2^SCAN-NL or RSX-r^2^SCAN-NL is very competitive^58^ with respect to other dispersion-corrected meta-GGA functionals such as SCAN-D3­(BJ), M06L-D3(0), or M11L-D3(0), for which values between 8–10 kcal mol^–1^ are found.

**Table 5:** WTMAD-4 (in kcal mol^–1^) for the Different Subsets and the Global GMTKN55 Database

		basic +	iso. +			
Functional	small	large	barriers	intramol. NCI	intermol. NCI	GMTKN55
RSX-r^2^SCAN	6.37	5.92	8.19	11.35	13.82	8.97
RSX-r^2^SCAN-NL	6.40	5.60	8.87	8.42	7.12	7.07
RSX-r^2^SCAN0	7.19	7.15	5.34	11.24	14.87	9.29
RSX-r^2^SCAN0-NL	7.45	7.13	6.04	6.91	7.01	7.03
RSX-r^2^SCAN-QIDH	6.01	5.57	4.15	5.92	8.77	6.29
RSX-r^2^SCAN-QIDH-NL	6.17	5.65	4.37	5.32	6.36	5.76
ωr^2^SCAN	6.95	6.66	5.91	12.09	16.54	9.70
ωr^2^SCAN-NL	6.40	6.26	6.18	6.77	5.41	6.19
ωPr^2^SCAN50	3.55	3.30	2.97	6.66	11.26	5.62
ωPr^2^SCAN50-NL	3.30	3.04	3.52	3.77	4.07	3.53

The global performance of the parametrized ωPr^2^SCAN50 (WTMAD-4 of 5.6 kcal mol^–1^) is slightly better than that of RSX-r^2^SCAN-QIDH (WTMAD-4 of 6.3 kcal mol^–1^), although the latter is more accurate for the set of intra- and intermolecular noncovalent interactions. The incorporation of the VV10 nonlocal correlation functional, thus giving rise to the RSX-r^2^SCAN-QIDH-NL or ωPr^2^SCAN50-NL models, further improves the WTMAD-4 results, particularly affecting the intra- and intermolecular noncovalent interactions. The final WTMAD-4 value for the latter is quite close to the best results available in the literature for double-hybrid functionals, for which WTMAD-4 values of 2–3 kcal mol^–1^ can be obtained.^58^

#### 3.3.5 A Note on the C60ISO Data Set

The C60ISO data set comprises isomers of C_60_ and their relative energies, which are of great interest for benchmarking computational methods^59–61^ and whose reference energies were very recently revisited.^62^ The authors of the latter study found discrepancies greater than 10 kcal mol^–1^ between previously calculated [at the DLPNO-CCSD­(T)/CBS level] and updated values [at the PNO-post-LCCSD­(T)/CBS level], which prompted us to reevaluate the performance of the different methods using these new reference values. Table 6 shows the impact of the updated reference values on the error metrics, which are systematically worse here since the updated reference isomerization energies are lower than the original values.

**Table 6:** MSD, MAD, and RMSD (All in kcal mol^–1^) Values for the Relative Energies of the C60ISO Data Set of Compounds

	Previous reference values			Updated reference values		
Functional	MSD	MAD	RMSD	MSD	MAD	RMSD
RSX-r^2^SCAN	8.19	8.19	8.67	12.90	12.90	14.41
RSX-r^2^SCAN-NL	7.74	7.74	8.14	12.45	12.45	13.87
RSX-r^2^SCAN0	13.78	13.78	14.85	18.50	18.50	20.62
RSX-r^2^SCAN0-NL	13.26	13.26	14.24	17.98	17.98	20.01
RSX-r^2^SCAN-QIDH	5.20	5.20	5.63	9.91	9.91	10.63
RSX-r^2^SCAN-QIDH-NL	5.06	5.06	5.49	9.77	9.77	10.46
ωr^2^SCAN	14.22	14.22	15.47	18.94	18.94	21.32
ωr^2^SCAN-NL	13.66	13.66	14.80	18.33	18.33	20.60
ωPr^2^SCAN50	3.26	3.26	3.52	7.98	7.98	8.79
ωPr^2^SCAN50-NL	2.91	2.91	3.18	7.58	7.58	8.32

The range-separated double-hybrid models, with or without dispersion corrections, led to the lowest values (around 8–10 kcal mol^–1^) for the set of functionals assessed here. The best values reported in the literature lie between 2 and 5 kcal mol^–1^ for double-hybrid functionals, although larger deviations (e.g., up to 18 kcal mol^–1^ with ωB2-PLYP) can also be found. The influence of the updated error metrics for C60ISO is negligible for WTMAD-2 (around 0.01–0.02 kcal mol^–1^) but can decrease WTMAD-4 by up to 0.1 kcal mol^–1^ due to their different formulations. Logically, if we look at the performance of methods exclusively for the “iso. + large” family (to which the C60ISO data set belongs) of the GMTKN55 database, the impact of the updated reference values is much higher, on the order of 0.5 kcal mol^–1^, which might be taken as a note of caution for the comparison and ranking of methods employing different reference values.

## 4 Conclusions

A new family of range-separated r^2^SCAN functionals was developed (named RSX-r^2^SCAN, RSX-r^2^SCAN0, and RSX-r^2^SCAN-QIDH) where the range-separation parameter (ω) was determined by a first-principles constraint; i.e., the model was forced to reproduce the energy of the hydrogen atom rather than performing any parametrized fitting for ω, thus aligning with the nonempirical philosophy of the original r^2^SCAN exchange-correlation model. The introduction of exchange range separation substantially reduces delocalization (self-interaction) errors, as was clearly shown by first studying the ionization potential of increasingly large He_*n*_ clusters, for which the size-dependent delocalization error is consistently reduced across the hierarchy of models. The largest improvement is obtained for the double-hybrid RSX-r^2^SCAN-QIDH functional, for which a delocalization error of around 1 eV was obtained after comparing the results for the smaller and larger clusters considered. Additionally, using the large GW100 data set of ionization potentials, the accuracy also systematically improved from the meta-GGA to the hybrid and finally to the double-hybrid level, with RSX-r^2^SCAN-QIDH achieving a MAD of only 0.14 eV.

Across the GMTKN55 database, which is widely used to compare the performance of DFAs, moving from RSX-r^2^SCAN to RSX-r^2^SCAN0 provides only modest improvements (except for reaction barrier heights), whereas RSX-r^2^SCAN-QIDH significantly lowers overall errors and performs consistently well for diverse chemical problems. This is also observed for subsets involving proton affinities, electron affinities, and especially the SIE4 × 4 benchmark, where RSX-r^2^SCAN-QIDH clearly outperforms the rest of the models for the latter subset. The newer WTMAD-4 metric was also used to compare and rank the different models assessed here. Although the relative rankings change somewhat compared with WTMAD-2, the RSX-r^2^SCAN functional remains very competitive. In particular, RSX-r^2^SCAN performs better than the empirically parametrized ωr^2^SCAN under WTMAD-4, while RSX-r^2^SCAN-QIDH achieves a WTMAD-4 value slightly higher than that achieved by ωPr^2^SCAN50. Once a nonlocal correction for dispersion is added, the RSX-r^2^SCAN-NL, RSX-r^2^SCAN0-NL, and RSX-r^2^SCAN-QIDH-NL variants reduce errors for intra- and intermolecular noncovalent interactions, thereby lowering the WTMAD values regardless of which variant of this metric used.

The results presented here show that the range-separated extension of the r^2^SCAN family can achieve an accuracy close to that of optimized functionals while avoiding any parameter fitting, whether automated or otherwise. Note also that a reevaluation of any reference value, as happened very recently with the C60ISO subset, might also alter the parametrization, which would not be the case for the models developed here. Among the proposed methods, RSX-r^2^SCAN-QIDH offers the best compromise between minimal empiricism and broad chemical accuracy, with its VV10-corrected version (i.e., RSX-r^2^SCAN-QIDH-NL) delivering reasonable performance for thermochemistry, kinetics, and noncovalent interactions.

## Supplementary Material





## Data Availability

The data underlying this study are available in the published article, the Supporting Information, and the open-access^63^ ioChem-BD^64^ repository via a database.^65^

## Notes

(i) The graphical comparison (RSX-r^2^SCAN-based family of functionals vs ωr^2^SCAN and ωPr^2^SCAN50) for the MAD of each subset within the families of properties of the GMTKN55 database provided by the different functionals assessed here; (ii) the graphical comparison (RSX-r^2^SCAN-based vs r^2^SCAN-based family of functionals) for the MAD of each subset of the GMTKN55 database; (iii) the raw data from calculations on He_*n*_ clusters; (iv) the raw data for the GW100 data set; (v) detailed and complete error metrics for each subset of the GMTKN55 database and for all the functionals developed here (PDF)
The raw data accompanying the calculations performed (XLSX)
