# Supplementary material for: A New Family of Nonempirical Range-Separated Functionals: RSX‑r2SCAN, RSX‑r2SCAN0, and RSX‑r2SCAN-QIDH
Source: J Chem Theory Comput. 2026 Oct 15;22(20):10721–31. doi: 10.1021/acs.jctc.6c01471 (PMC13718555; doi:10.1021/acs.jctc.6c01471)
Supplement: Supplementary file 1 [file ct6c01471_si_001.pdf]

–Supplementary Material–

A new family of non-empirical range-separated functionals: RSX-r^{2}SCAN, RSX-r^{2}SCAN0, and RSX-r^{2}SCAN-QIDH

P. Maiz-Pastor[a], E. Brémond[b,c], A.J. Pérez-Jiménez[a], C. Adamo[d], and J. C. Sancho-García[a*]

[a] Department of Physical Chemistry,
University of Alicante,
E-03080 Alicante, Spain

[b] Université Paris Cité, ITODYS, CNRS,
F-75006 Paris, France

[c] Institut Universitaire de France, 103 Boulevard Saint Michel,
F-75005 Paris, France

[d] Chimie ParisTech, PSL Research University, CNRS,
Institute of Chemistry for Life and Health Sciences (i-CLeHS), UMR 8060,
F-75005 Paris, France

September 7, 2026

*jc.sancho@ua.es

1 Comparison between RSX-r^{2}SCAN-based functionals and ωr^{2}SCAN and ωPr^{2}SCAN50

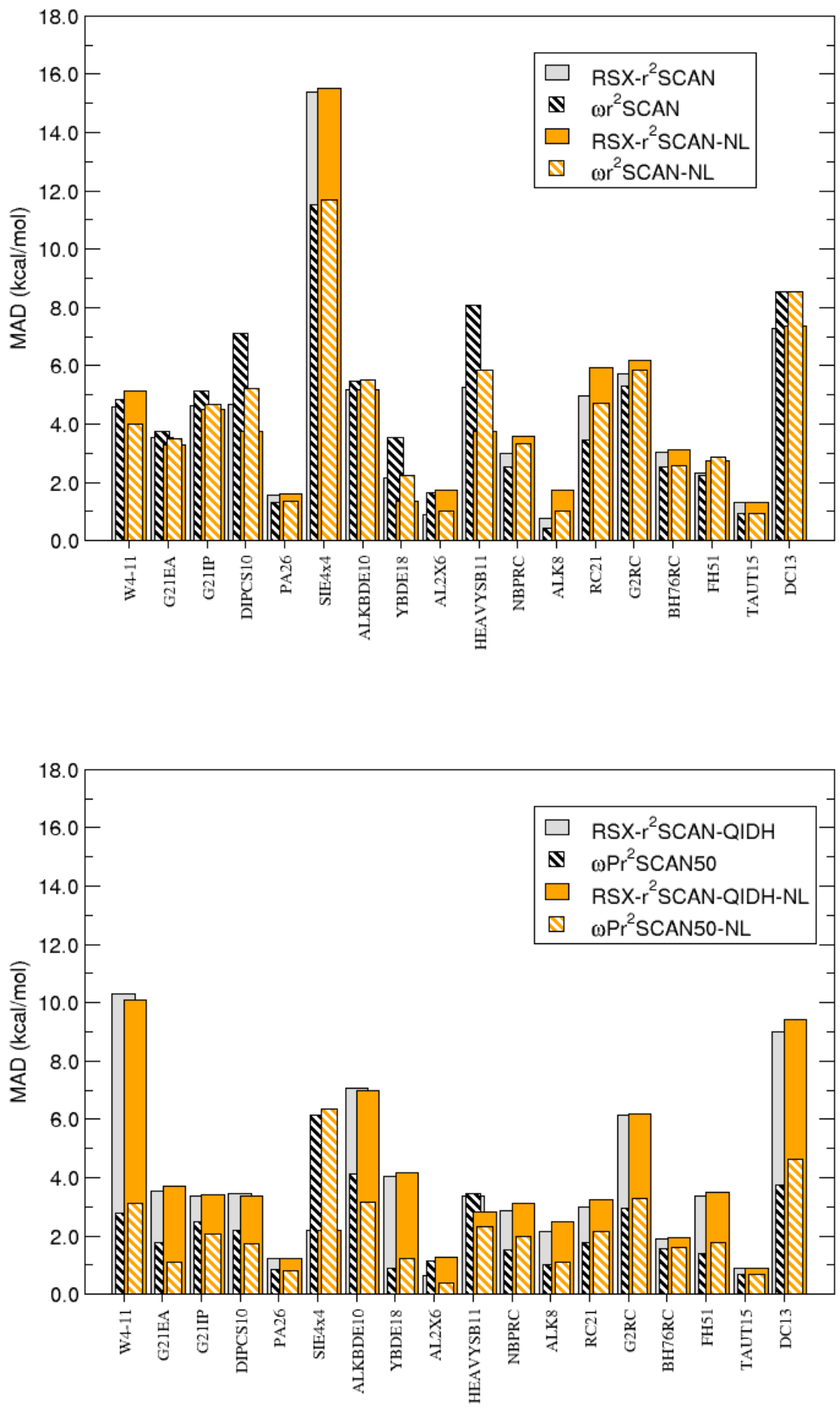

Fig. S1: MAD for each subset of the families of properties of the GMTKN55 database: basic properties and reaction energies for small systems.

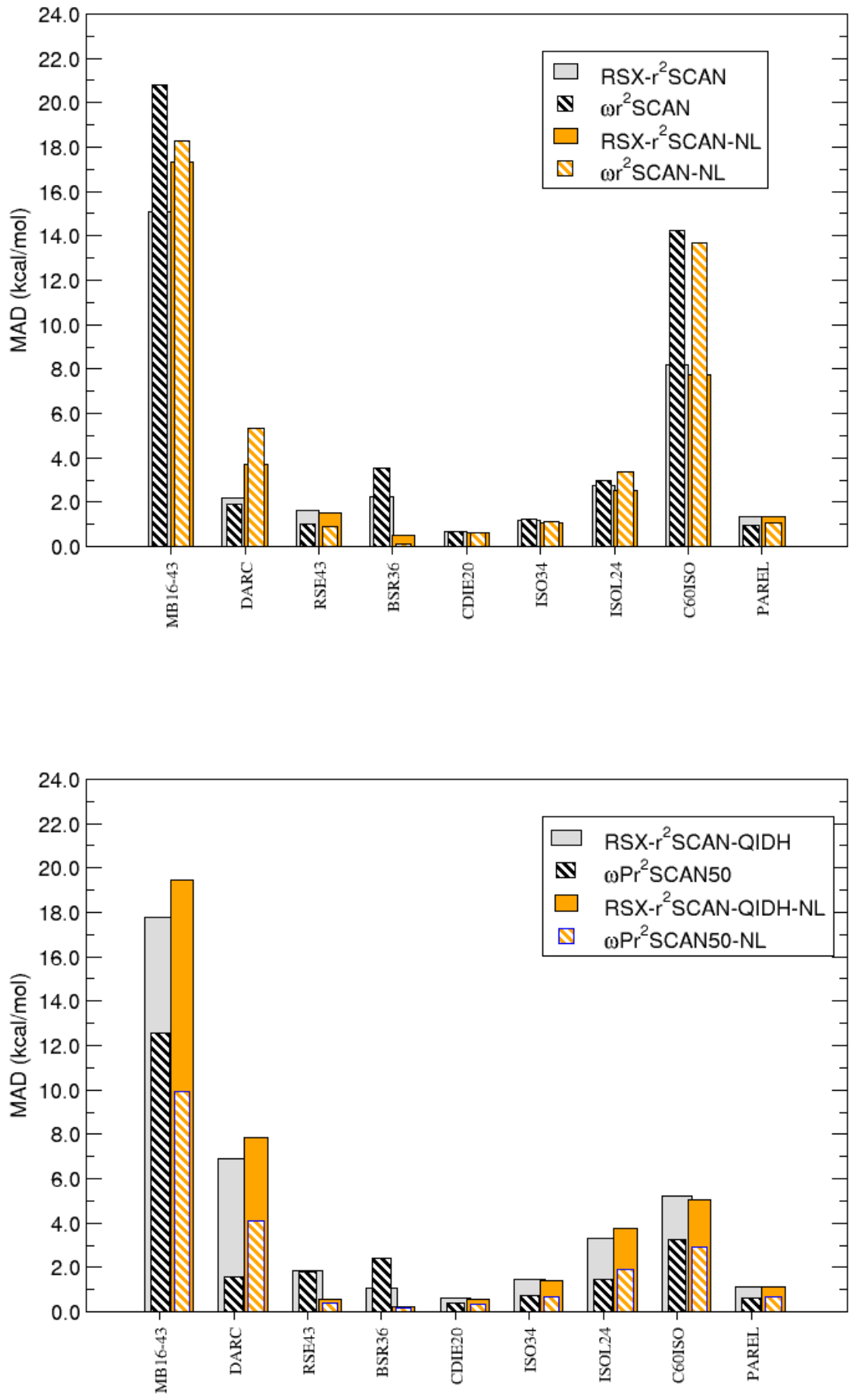

Fig. S2: MAD for each subset of the families of properties of the GMTKN55 database: isomerisation and reaction energies for large systems.

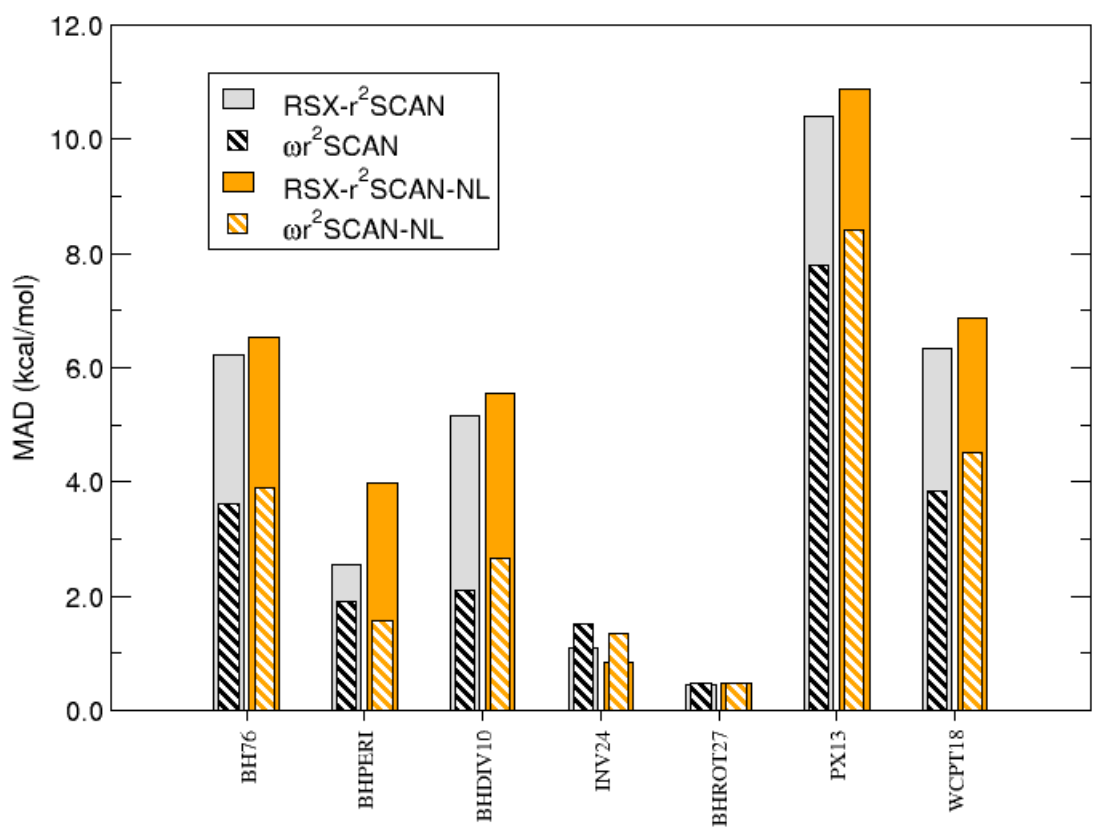

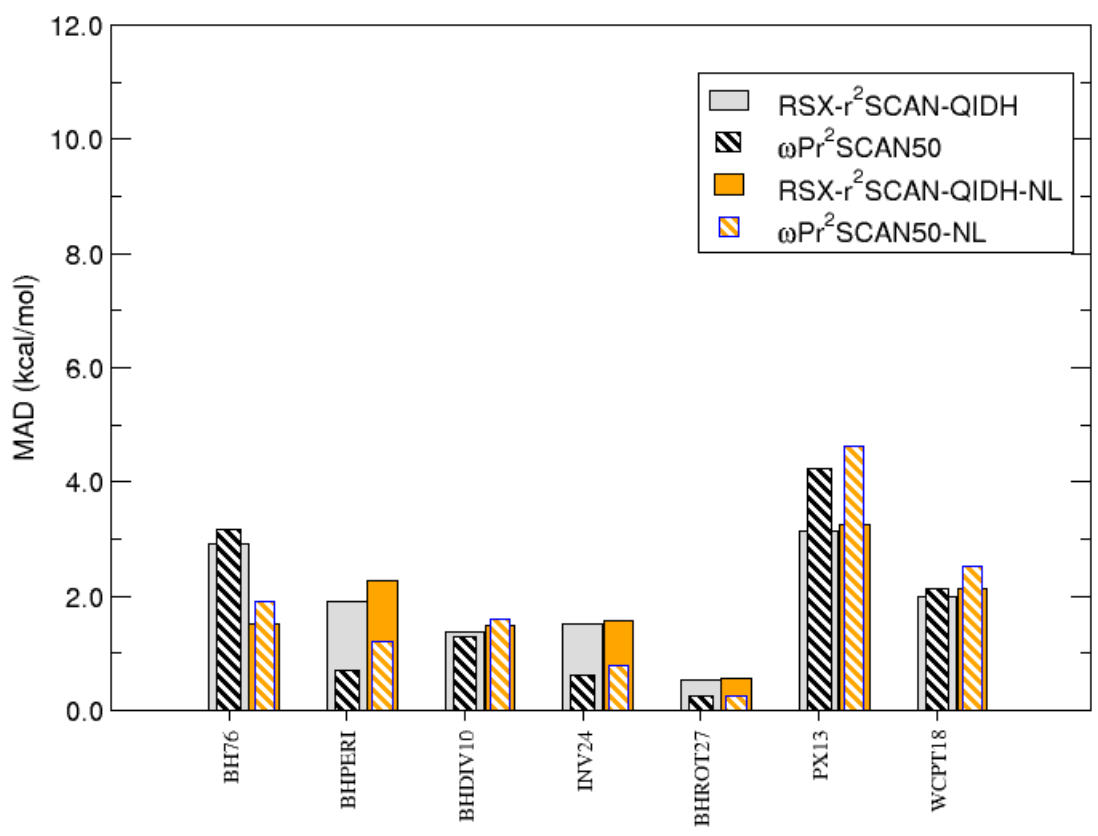

Fig. S3: MAD for each subset of the families of properties of the GMTKN55 database: reaction barrier heights.

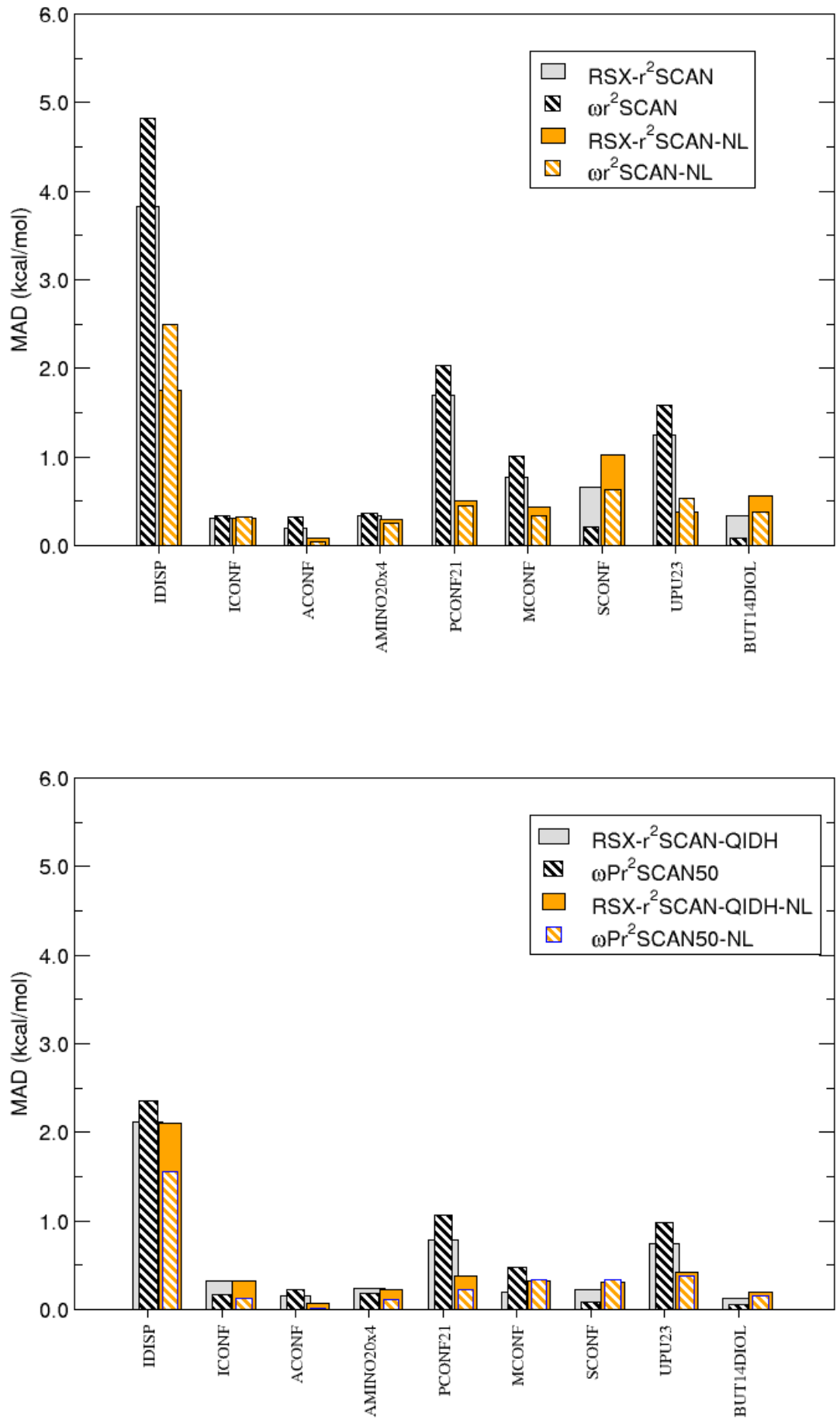

Fig. S4: MAD for each subset of the families of properties of the GMTKN55 database: intramolecular non-covalent interactions.

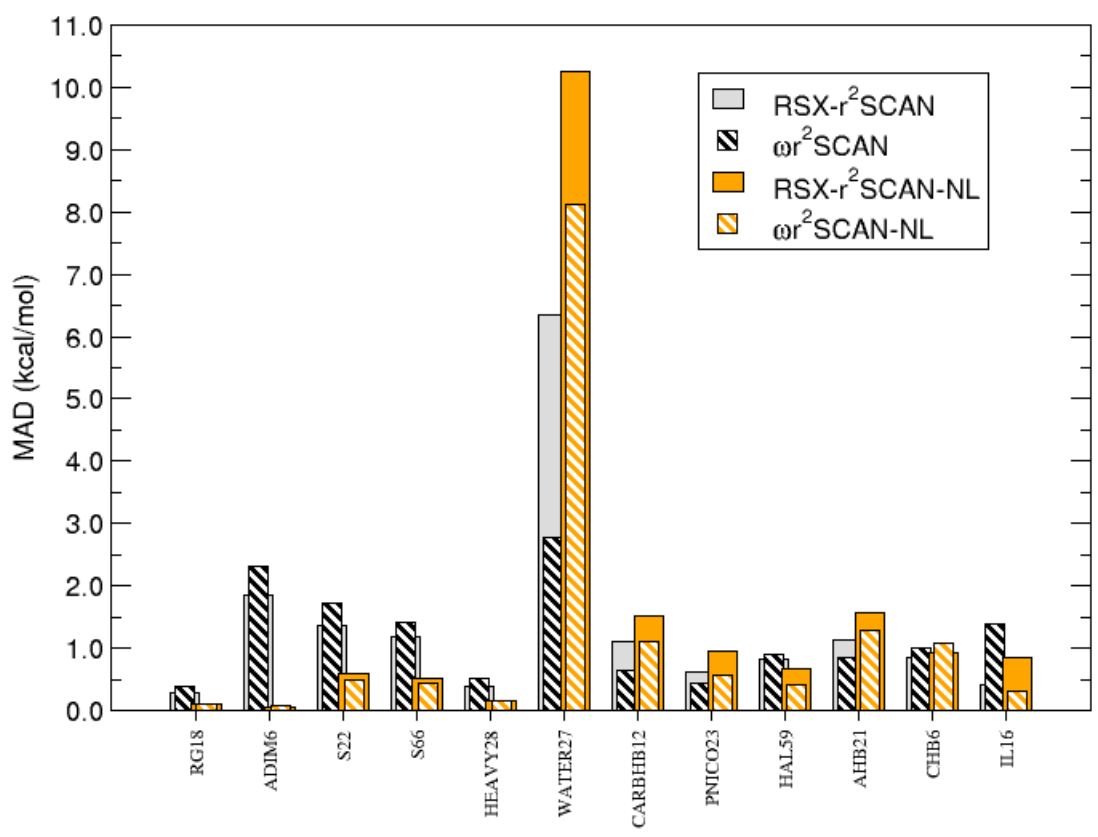

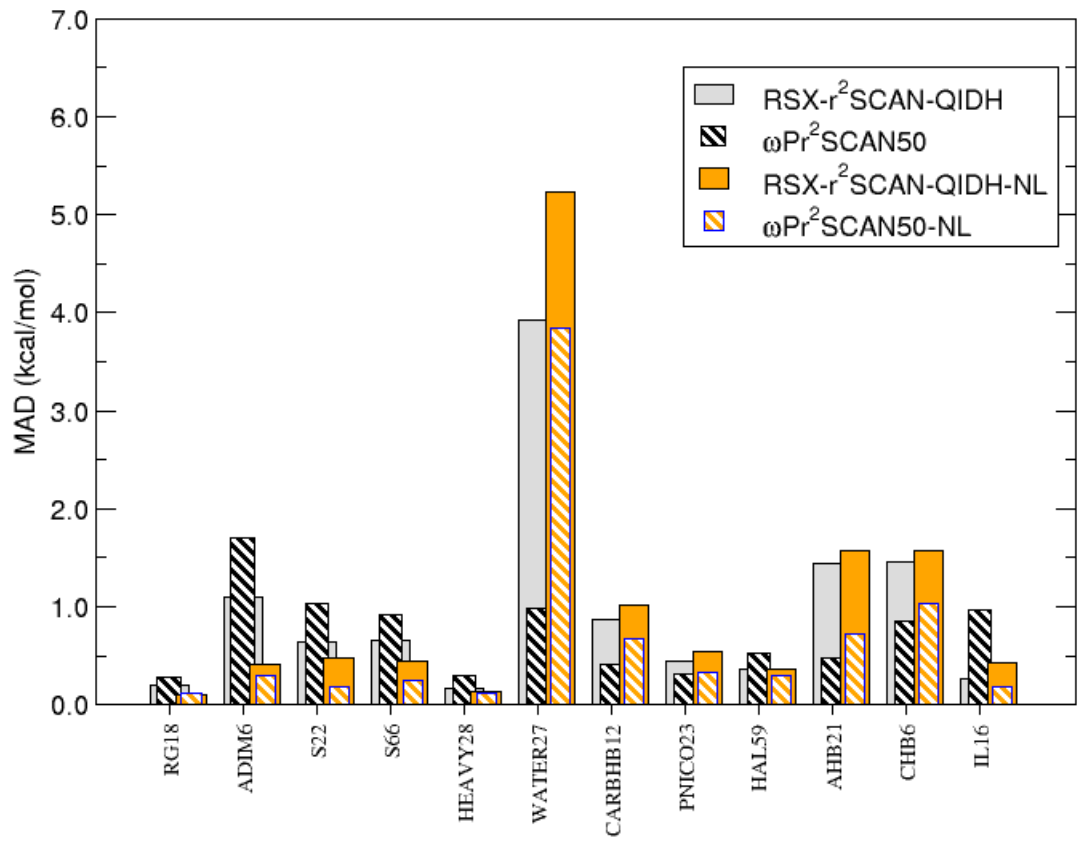

Fig. S5: MAD for each subset of the families of properties of the GMTKN55 database: intermolecular non-covalent interactions.

2 Comparison between RSX-r^{2}SCAN-based and r^{2}SCAN-based functionals

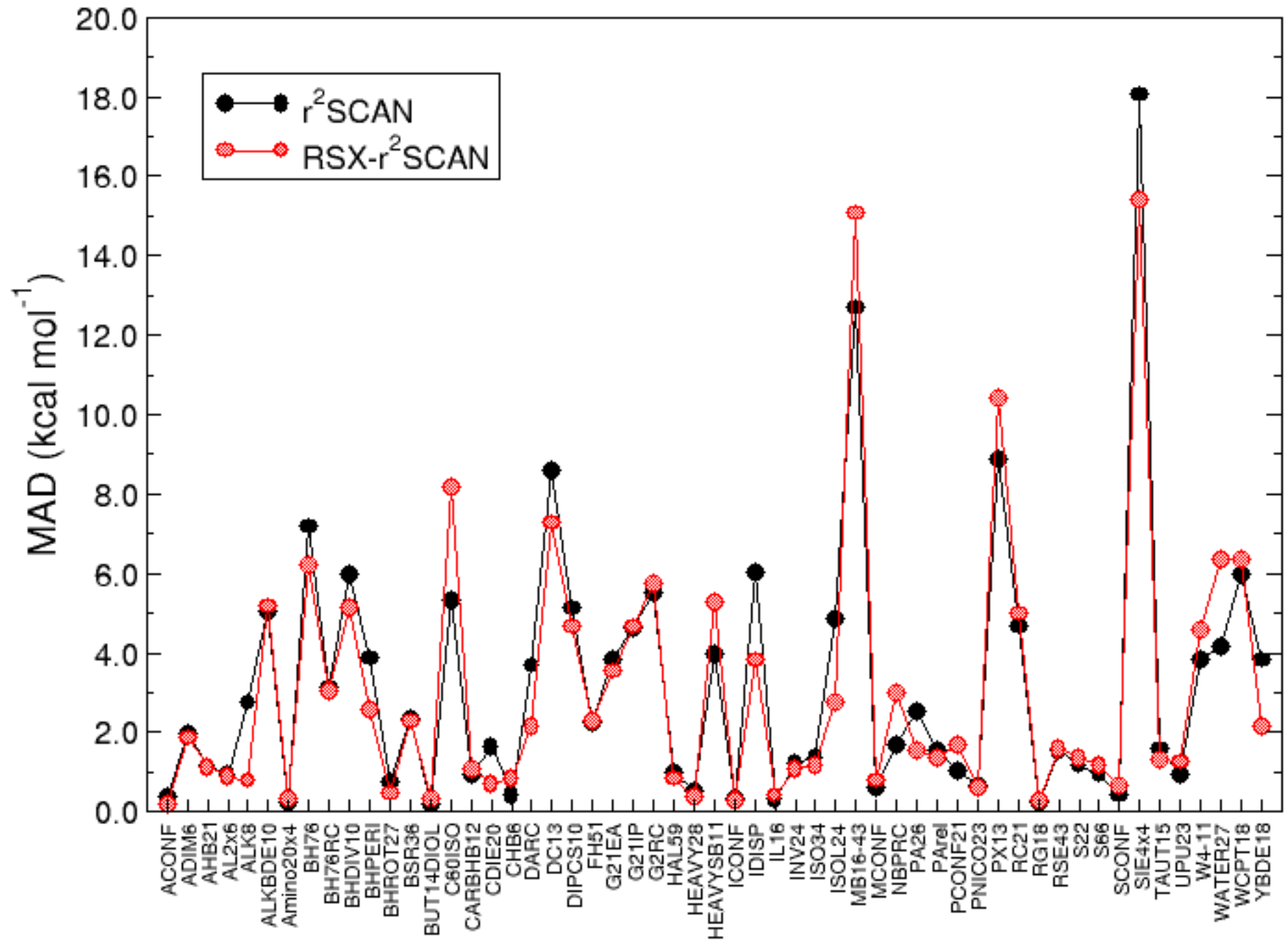

Fig. S6: MAD for each subset of the GMTKN55 database: RSX-r^{2}SCAN vs. r^{2}SCAN.

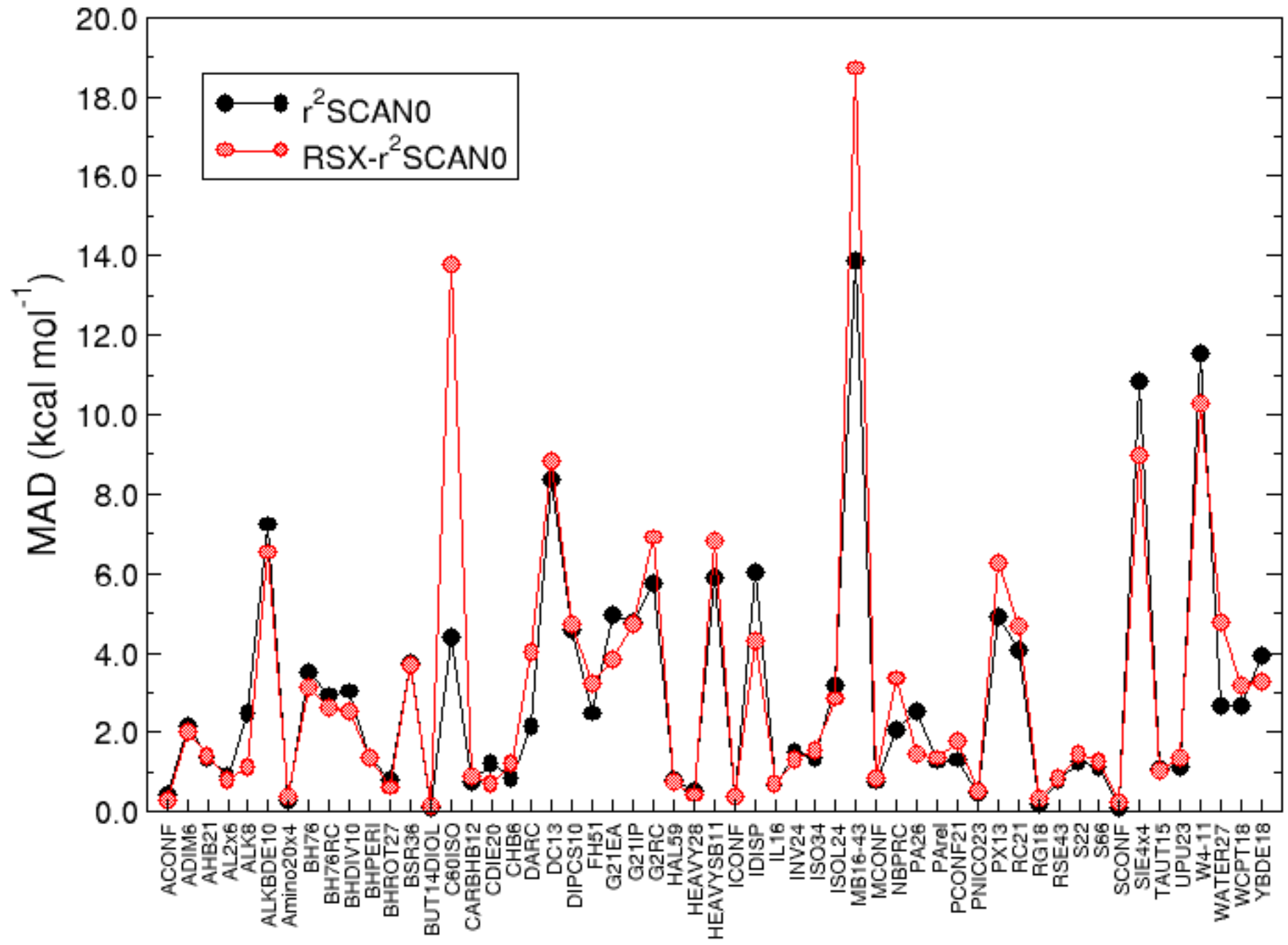

Fig. S7: MAD for each subset of the GMTKN55 database: RSX-r^{2}SCAN0 vs. r^{2}SCAN0.

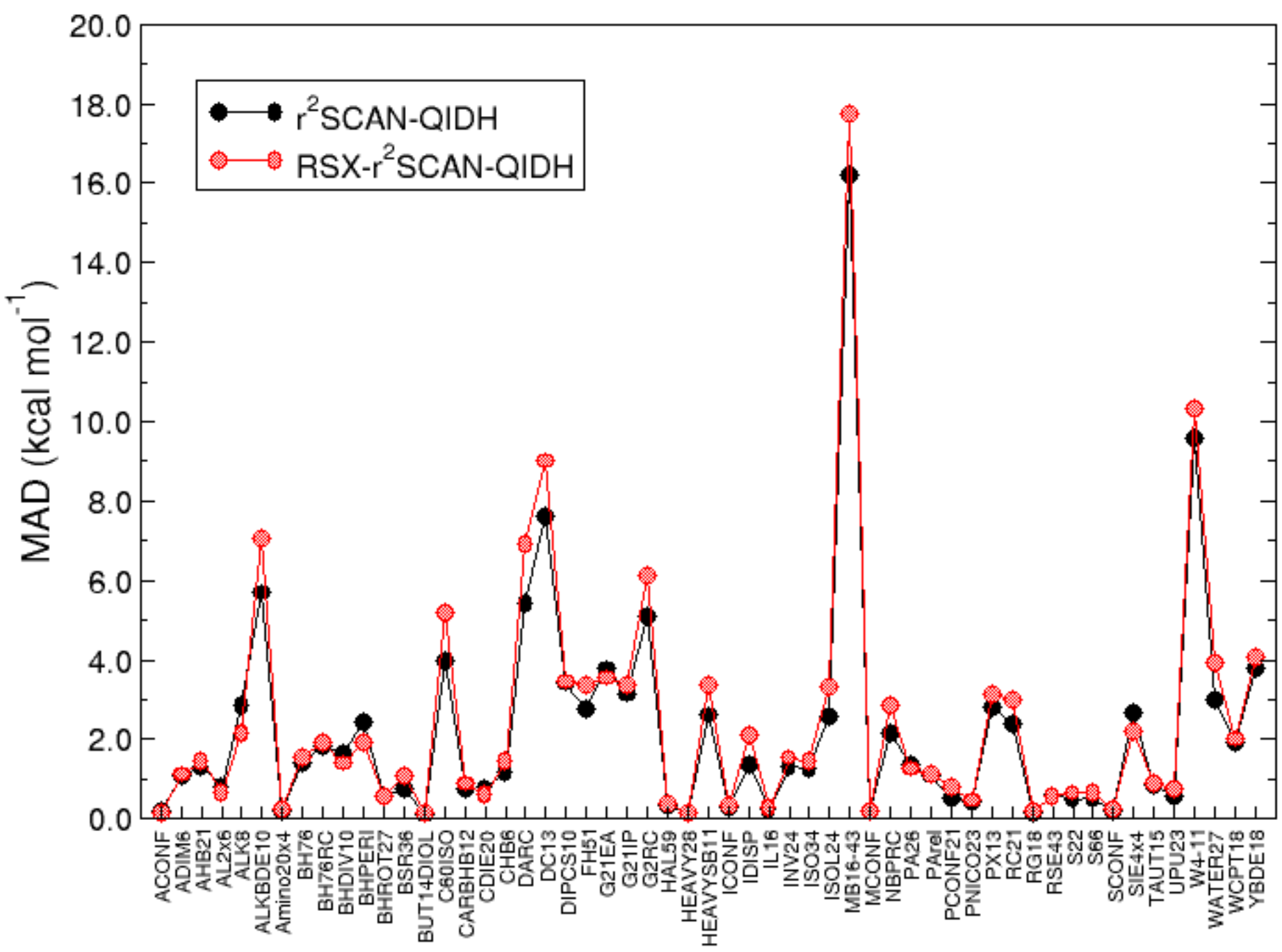

Fig. S8: MAD for each subset of the GMTKN55 database: RSX-r^{2}SCAN-QIDH vs. r^{2}SCAN-QIDH.

3 The RSX-DFA-QIDH model

The exchange component of the RSX-DFA-QIDH model, where DFA stands for Density Functional Approximation, corresponds to the following expression:

$$\begin{aligned} E_x^{\mathrm{RSX-DFA-QIDH}}[\rho(\mathbf{r})] &= \underbrace{E_x^{\mathrm{DFA}} - \left[a_x E_x^{\mathrm{DFA}} + (1-a_x)\, E_{x,\omega}^{\mathrm{DFA}}\right]}_{\text{SR = short-range}} \\ &+ \underbrace{a_x E_x^{\mathrm{EXX}} + (1-a_x)\, E_{x,\omega}^{\mathrm{EXX}}}_{\text{LR = long-range}}, \end{aligned}$$

where E_x^{DFA} and E_x^{EXX} are, respectively, the semilocal DFA and EXact-eXchange (EXX) energies, with $E_{x,\omega}^{\mathrm{DFA}}$ and $E_{x,\omega}^{\mathrm{EXX}}$ their respective Coulomb-attenuated variants. The RSX-DFA-QIDH expression recovers the DFA-QIDH model in the short-range regime; i.e., when $|\mathbf{r}-\mathbf{r}'|$ tends to vanish. The extension to include the correlation term is simply:

$$E_{xc}^{\mathrm{RSX-DFA-QIDH}}[\rho(\mathbf{r})] = E_x^{\mathrm{RSX-DFA-QIDH}} + a_c E_c^{\mathrm{PT2}} + (1-a_c)\, E_c^{\mathrm{DFA}},$$

where E_c^{DFA} and E_c^{PT2} are respectively, the semilocal DFA and 2nd.-order Perturbation Theory energies. The E_c^{PT2} term can be also splitted into a same-spin (ss) and opposite-spin (os) contribution:

$$E_c^{\mathrm{PT2}} = c_{ss} E_c^{ss-\mathrm{PT2}} + c_{os} E_c^{os-\mathrm{PT2}}.$$
